# Medical Miscellanies

**Published:** 1816-09

**Authors:** 


### PART IV.

### MEDICAL MISCELLANIES.

### MORBID ANATOMY.

(Selected and abridged from MORGAGNI.)

WITH COMMENTARIES.

(Continued from our last, page 167.)

"Wherever Morbid Anatomy has been steadily adhered to as the foundation of observation and reasoning in our science, that science has been progressive: it has been interrupted in its course, even amidst the most brilliant reputation of its professors, wherever men have arisen, whose inventive powers have led them to reject, rather than to seek the aid of Anatomy."—*C. Bell, on the Diseases of the Urethra.*

CASE 7. A sexagenarian, who was accustomed to drink freely of wine, and who had frequently fallen down with vertigo, was found, immediately after a hearty dinner, lying dead on the ground, his upper extremities much contracted, and the fæces alvi emitted. He had been immediately before this in excellent health.

*Sectio Cadaveris.* The cranium being sawed through, and the dura mater perforated anteriorly, a limpid water gushed from between this and the pia mater. The latter contained, in the interstices of its vessels, a gelatinous fluid of a palish colour. In the lateral ventricles, some of the glands of the plexus choroides were so turgid as to equal the largest lentils in size. In the right ventricle were two grumous concretions of blood. In both sides of the cerebellum, the blood was so coagulated as to resemble one solid polypous body. About these concretions the cerebellum had a soft consistence, like rotten fruit.

Remarks. This last case, and indeed every day's experience, shews how much people, disposed to vertiginous affections, should beware of what they consider a state of unusual good health, or, as Celsus expresses it, " *Suspecta habere sua bona debent.*"

The foregoing cases are all from Valsalva, and occupy the first Letter on Sanguineous Apoplexy, or second in the work. The third, or following Letter, contains the experience of Morgagni himself.

*Lett. III. On Sanguineous Apoplexy, in Continuation.*

Case 8. A Venetian woman, ætat. 50, of a florid countenance, large stature, and full habit of body, was long subject to violent colicky pains; and ever since her last pregnancy, had grown very prominent in her belly, so as to be incommoded much in the execution of her domestic offices. On these accounts, she said she could not drink her wine diluted with water; she therefore drank it in a pure state, and *pretty copiously.* She now got slothful, dull, and inclined to sleep. For some days previously to the event about to be described, she complained of a troublesome noise in her head. At length, about the third hour of the night, she was seized with a pain in the *right* temple and eye, and, while attempting to sit down in a chair, she became apoplectic, and fell on her *left* side, the motion of the right arm continuing for an hour afterwards. By a feeble action of vomiting, she threw up the wine she had drunk in the day. Stertor now came on, and she died in a few hours.

*Sectio Cadaveris.* The whole right hemisphere of the brain was covered with a layer of coagulated blood effused beneath the dura mater. This lamina being removed, the vessels of the pia mater, on both hemispheres, were found turgid with blood. From the exterior surface of the right hemisphere, two or three foramina led into a large cavity, formed in the medullary substance of the same hemisphere, between the external side and the lateral ventricle, two fingers' breadth in width, and six or more in length. The parietes of this cavity were eroded; it was full of grumous blood, and communicated with the right ventricle posteriorly. By this communication some blood had passed into the ventricle, and over into the other one, by breaking down the septum lucidum. A lamina of blood was also found below the transverse process of the dura mater, covering the whole cerebellum, of moderate thickness. In the spinal tube, as far as could be seen, a quantity of blood was extravasated round the spinal marrow.

Case 9. A stout muscular porter, 40 years of age, who said he had never been ill in his life, died suddenly, at the fourth hour of the night, of apoplexy. His body was publicly and accurately examined by Morgagni himself.

*Sectio Cadaveris. Abdomen.* Colon very much contracted, except at each extremity, where it was inflated with wind. Ileum thickened and sphacelated at its extremity. Liver, hard; in colour, externally, like reddish marble, variegated with white. Internally, this viscus appeared as though it were boiled. Gall-bladder contained a deep green, almost black, bile, with several black calculi, having irregular and jagged surfaces, as if broken into pieces by accident. *Thorax.* Some of the valves of the right side of the heart discovered incipient points of ossification. *Head.* Extravasation of blood, under the pia mater, on the right hemisphere of the brain, with turgidity of vessels. In the left hemisphere, a great cavern lay hid, formed in the middle of its substance, and hollowed out from the medullary part longitudinally; which cavern was full of the most black and half concreted blood. The vessels of this side were much less turgid than in the other. The internal parietes of this cavity were lacerated, and communicated with both ventricles, which were full of serum.

Remarks. It is much to be feared, as well as regretted, that in the dissection of apoplectic victims, the *immediate* seat of the disease, and the *immediate* cause of death, have too much engrossed the anatomist's attention, while the remote and original sources were not sufficiently explored. Modern physicians have traced many affections of the head to derangement of function or structure in the viscera of the abdomen, and particularly the liver. Hydrencephalus in youth, and apoplexy in age, are probably both connected with difficult transmission of blood through the system of the vena portarum. Morgagni here sneers at the opinion of Fred. Hoffman, who, on finding biliary calculi in the defunct of apoplexy, attempts to explain the manner in which the *latter* disease is brought on, by supposing, that the spasmodic contractions, induced in the belly and neighbouring vessels by gall-stones, might occasion a greater quantity of blood to be retained in the upper parts of the body. Although Hoffman's *modus operandi* may not now be allowed, yet the connexion of hepatic derangement with increased determination of blood to the head, is every day more clearly discerned and acknowledged.

Case 10. Anthony Tita, author of the "*Catalogus Plantarum*," died suddenly at Padua, in May 1729. The weather was then very hot, after long-continued rains and cold; and numbers of people were swept away with only a few hours' notice. Tita was 73 years of age, still robust and brawny; square figure, and somewhat fat. He was in the habit of exposing himself much to the sun; and to drink pretty freely, though not to drunkenness, of undiluted wines. He had been troubled, for some years, with ophthalmic inflammation, and lately complained of *fulness in his head*. On the 4th of May, the sun being unusually hot, having spent the whole day in the open air, and supped in the evening as usual, he suddenly cried out that he was very ill, lost the use of his tongue, and became hemiplegic on the left side. Morgagni immediately visited him. His senses were perfect; colour, respiration, and temperature, natural; pulse full and strong; complained of no pain, but seemed drowsy. Venæsection from the *right* arm was performed; a smart purging glyster administered, and ol. succini applied to the olfactories. Morgagni having retired, one of Tita's own physicians came in, and thought proper to give the patient an emetic; soon after the operation of which, a still more violent attack was experienced, with stertor and convulsions, which ended next morning in death.

*Sectio Cadaveris.* The dura mater adhered firmly to the skull. Its vessels (excepting the sup. long. sinus) were black from turgidity. Vessels of pia mater turgid also. *Right* lateral ventricle distended with blood of a black, coagulated appearance, and sufficient to fill the shell of a hen's egg. In the three other ventricles, there was fluid blood mixed with serum. The brain was sound, and the substance of the hemispheres entire, so that it did not appear from whence the blood and serum could have issued. Towards the posterior part of each lateral ventricle, especially of the right, the plexus choroides had vesicles, the size of large grapes, full of water.

Remarks. The propriety of exhibiting emetics in sanguineous apoplexy has occasioned much discussion, and even bitter and disgraceful litigation. That the *straining* during vomiting drives the blood with increased impetus through the arterial and capillary system to all parts of the body, and the head in particular, is most indubitable. This is evinced by the redness and swelling of the face, and accelerated velocity with which blood will flow from an orifice in a vein during the action of vomiting, as we have lately seen in some remarkable instances. It is stated, however, as the opinion of Dr. Parr, in the Lond. Med. Dict. that it is doubtful "whether, in every hæmorrhage, vomiting is not as useful, *by deriving to the surface*, as injurious from any other effect." We would just observe on this passage, that, in deriving blood to the surface from the interior, it *must* be driven through the capillary system into the veins, and consequently its course must be accelerated through the bleeding vessels, *wherever they are situated.* Surely then, to unload the vascular system itself, and particularly the vessels of the brain, by blood-letting, while determinations of blood to all, or any other parts, are solicited by those means which do not quicken, or much disturb, the general circulation, as purging, blistering, sinapisms, &c. are preferable to the hazardous experiment of vomiting, and its usual concomitant, straining. How often do we see apoplexy brought on by stooping to buckle the shoe, lifting a heavy weight, straining to evacuate a costive stool, &c.? in all which the blood is driven violently to the head through the arteries, while its return through the veins from thence is greatly impeded.

Case 11. *Ossified falx.* A man, advanced in years, had died of apoplexy of a few days' standing. All that could be learnt was, that while he lay in the apoplectic paroxysm, he had a strong pulse, but not difficult respiration.

*Sectio Cadaveris.* Abdominal viscera natural. In the thorax were universal adhesions between the pleura pulmonalis and p. costalis. The heart was so covered with fat, that, on looking on it anteriorly, nothing but adipose substance was visible. The valve of the coronary vein was every where fixed, and pierced through with little foramina. The left vertebral artery originated from the curve of the aorta itself, instead of the subclavian. Half a pound of blood was extravasated between the dura pia mater of the left side. Within the duplicature of the falx, and near to the lower edge, was an ossification more than three fingers' breadth in length, and an inch and a half in breadth, moderately, yet unequally thick, and lying longitudinally. On both right and left surfaces it had large protuberances like studs or bosses, covered over by the membrane of the falx.

CASE 12. A youth of Bologna, 14 years of age, much troubled with worms, and accustomed, on the slightest exertion, or when sitting near the fire, to profuse nasal hæmorrhage, and being also addicted to the abuse of spirituous liquors, changed suddenly, and without any apparent cause, from a lively and brisk state to that of hebetude and dulness. After a few days, and having conversed, one morning, with more than usual vivacity, he was found, in the afternoon, extended on his bed, upon which he had vomited; compressing his head between his hands, as though he felt great pain there, but without the power of speech, and very soon of motion. A physician ordered venæsection, which seemed to relieve him a little, and volatile ammonia was applied to his nostrils; but he soon relapsed into his former torpor, though he appeared sensible of what was said. His pulse was low and intermitting. His respiration bad, and he had some foam at the mouth. Cupping-glasses were applied to the back, but he was insensible of them. When applied to the thighs, he cried out, though inarticulately, and tried to remove them. No amendment, however, taking place, he died about the ninth hour of the night.

*Sectio Cadaveris.* The head only was examined. In the lateral and third ventricles some serum was found. Under the cerebellum, the substance of which was softer than natural, and nearly in the middle, two spoonfuls of black coagulated blood were found.

CASE 13. Peter Fasolati, an engraver at Padua, ætat. 62, full habit, and always very healthy, retired to bed after a hearty supper, but was found, in two hours, not only quite dead, but cold, and stretched out in the position which he first assumed on lying down.

*Sectio Cadaveris.* While sawing through and removing the skull-cap much blood was discharged. No extravasated blood, however, appeared within the skull, nor in the substance of the cerebrum or cerebellum, both to the touch being perfectly natural. A small quantity of limpid water was found in the lateral ventricles, and some also seemed to flow from the sides of the cerebellum, or probably issued from the theca vertebralis. The whole *vascular* system, however, of the brain, was distended with fluid blood, in such a manner as I had never before witnessed. Even some small vessels, which usually are scarcely perceptible, were extremely large and turgid. In the thorax were some pleural adhesions, but no disease of structure in the lungs. In the pericardium was some tinged water, but not much. The heart was large, and its proper vessels and auricles turgid with blood, which came forth black and grumous. The blood was also black and grumous in the ventricles of the heart, but not in quantity. The valvula mitralis of the right ventricle was white, and harder than usual; as were some of the semilunar valves, their membranous parts having assumed a ligamentous nature. On the exterior surface of the right auricle were some white spots. The aorta and other vessels were healthy.

REMARKS. That apoplexy is frequently produced by *turgescence* of the vessels alone, was believed in ancient times as well as modern. " By this means (says Galen) apoplexies are brought on, to wit, by much blood rushing tumultuously into the principle of animation." (Apud Salium de Affect. Part.) It is indeed reasonable to suppose, that in the majority of apoplectic recoveries, *congestion* only had taken place in the vessels of the brain. But if congestion give rise to the more favourable cases, it appears capable of producing the most desperate and instantaneously mortal ones also. If a sudden and great determination of blood takes place to the head, and the vessels are not unloaded, either by artificial means, or by the rupture of their coats, or effusion of their serous contents, it is quite evident that the whole substance of the cerebrum and cerebellum becomes compressed, and the functions of life destroyed. And indeed it would appear, that in such cases, rupture or effusion is an effort of nature to procrastinate, if not save, the life of the patient. Rupture and effusion are only partial evils, and to a certain extent, and in certain portions of the brain are compatible with life, for some time at least; but general compression, to a certain degree, on the cerebrum and *cerebellum*, extinguishes it at once.

We may probably, in this way, account for the number of sudden deaths, in the night, which we continually hear of. A *gourman*, whose vascular system is highly plethoric from immoderate eating and little exercise, goes to bed after a *hearty supper*, and falls asleep. The loaded stomach now presses on the descending aorta, and a disproportion of blood is thrown through the carotids and vertebrals to the brain. If the vessels give way, and either blood or water be poured out, apoplexy takes place, and the family are roused by the struggles or stertor of the patient. But if the vessels are too firm, then a general compression on the *whole* sensorium extinguishes the vital spark in a few minutes, and the man, who went to sleep in perfect health, is found, by his astonished friends, a cold and lifeless corpse in the morning!

( To be continued. )

*Case of Disease simulating Pregnancy.*—A lady, aged forty-seven, tall and of strong constitution, had always menstruated regularly. Married at the age of fifteen, she had borne four children (the first at the age of sixteen, the last at that of twenty-seven) and miscarried five times, at different periods of gestation, from her twenty-seventh to her forty-second year. At forty-six, the menses, which till then had invariably appeared in the beginning of each month, did not take place during September 1814: and, soon afterwards, she experienced all the usual symptoms of pregnancy. At three months, I examined her without being able to affirm that she was pregnant. Four months after the suppression of the menses, the abdomen had acquired the developement commonly presented at this period of gestation. My patient had then all the symptoms which usher in abortion; pains shooting from the loins below the navel, and terminating at the anus, bloody discharge from the vulva, &c. I prescribed absolute rest in the horizontal posture and diluent drinks; I also performed venæsection, indicated by the hardness of the pulse, redness of the face, and weight of the head. Health was speedily restored by these means. Between the fourth and fifth months, she distinctly felt the motions of the child. About the seventh month, these motions not having been felt for some days, and my patient being anxious to know if she were really pregnant, I made a second examination; the results of which were as follows: The abdomen was quite as voluminous as it commonly is at this period of pregnancy: it was, moreover, tense, sensible on pressure; and the superior part of an oblong mass, which I took for the fundus uteri, rose nearly an inch and a half beyond the umbilicus. Introducing a finger into the vagina, I found the cervix uteri, and organ itself, of which I could touch a part of the posterior surface, in a natural state. Hereupon, I asserted that my patient was not pregnant, without, however, being able to explain the extraordinary increase in size of her abdomen. On the day after examination, motions, resembling those of the fœtus, were renewed; which confirmed my patient in the idea that, notwithstanding my assertion to the contrary, she was pregnant. Had the abdomen been at all unequally developed, I should have suspected the existence of extra-uterine pregnancy. Between the eighth and ninth months, the sensation of motions was stronger than ever. The breasts were very large, and a milky moisture oozed from them. Five days after, my patient felt severe pains in the kidnies, and had a slight discharge. Examination was impracticable. I had recourse to the means employed more than four months before. On the first and second days after the bleeding, more pain; the linen constantly a little stained with red. On the third, at nine in the morning, my patient called upon me, to say that she *had lain in without bearing children.* This was her expression. In fact, her breasts were shrunk; her abdomen reduced suddenly more than half, and very flaccid; and all this had taken place without any discharge of blood, alvine evacuation, excretion of urine or sweat, or the issue of flatus or water from the uterus. From this time, the lady recovered surprizingly. She experienced only a slight oppression in the epigastrium. Her menses re-appeared on the 15th of June, 14th of July, and 17th of August, and have continued as before the suppression. *Dr. Claudon, Journal de Médecine par Leroux. Octobre*, 1815.

*Quadruple Vision.*—A woman, somewhat above 30 years old, after a hard labour, combined with a violent hæmorrhagia uteri, saw every object fourfold. On account of her general debility and poorness of blood, a nourishing regimen and the following medicines were prescribed. ℞. Extract salicis ℥ ß dissolve in aqua cinnamomi sine vino ℥iij admisce; syrupi aurantii ℥i; naphthae aceti gt. xl; cap. cochl. j larg. 2da qq hor. ℞. Spirit æther comp. ℥ ß; olei cajeput. ʒ ß; M illinat. superciliis temporib. mane et vespere. After a fortnight's use of these remedies she recovered entirely, and saw objects singly.

*Case of fractured Bones in a new-born Infant.*—A woman, 30 years of age, of a healthy and good constitution, was, after a pregnancy of full nine months, in February 1803, naturally delivered of an apparently strong, middle sized female child. The woman this time had as easy a labour as she had had twice before: But very rare phænomena occurred in the child, viz. that the processes of the bones were broken, all along in the same place, as if external violence had been the cause of it. The shoulder-blades alone were entire. The internal lesions could not be discovered by external examination, and only on moving the parts, a cracking noise was heard, and the fractures felt. The child died after seven hours, and every one of the relations heard after the death the cracking of the broken bones. The mother on being questioned, whether she had not suffered some particular accident whilst pregnant, such as spasms, frights, &c. replied that nothing of the kind had ailed her, except a slight cough, which had given her but little inconvenience. In March 1805, she was delivered of a boy, who continues well up to this day, and has not the least symptom of bodily defect. The woman also is quite well, and is again gone four months with child.—*From the Giornal della Soc. Med. Chir. di Parma, by Surgeon Cetoni, of the Civil Hospital.*

*Easy Method of stopping too violent bleedings after the application of Leeches*—The hæmorrhage, after the application of leeches, is not owing alone to the opening of the small cuticular vessels, but also to an active congestion of blood from all the neighbouring vessels towards the wounded part, arising from the slow sucking of the leeches. In this manner, the bleeding is kept up for some time after the leeches have dropped off, and thus proves beneficial in most cases. In some few cases, however, when the bleeding continues too long, it becomes embarrassing, and sometimes, though rarely, if the blood is not disposed to coagulate, may even prove dangerous. Styptic applications, gum arabic, and tinder, often will not stop the bleeding, sufficient pressure can not in some parts be applied, and where it is applicable, it must be continued so long as to become very troublesome to the patient. Dr. Autenrieth, therefore recommends to fill up the small orifices; for which purpose he advises two or three threads of lint to be twisted between the fingers, and then to be screwed into the orifice as far as possible, the remaining part to be flattened and to be pressed to the wound in the form of a cake, which in a few moments will have the desired effect.

*Mode of tying Arteries at Acheen in Sumatra.*—Culprits are here punished according to the enormity of the offence, by cutting, or rather chopping off a hand or foot. To restrain the hæmorrhage, and at the same time serve as a wooden leg, (when the lower extremity is amputated) the following apparatus is provided. A bamboo cane is prepared, ready suited to the size and length of the culprits leg; the hollow of which cane is nearly filled with heated *dammer*, (a resinous kind of substance harder than pitch) and the instant the punishment is inflicted by lopping off the limb, the bleeding stump is thrust into the heated resin within the bamboo, which, as it cools, becomes fixed; and thus, if the victim lives, he is provided with a bamboo jury-leg to stump about upon.

*Lieut. Harriot's Travels.*

Mr. Battley, of Cripplegate, having decomposed Opium, and laid its component parts before the College of Physicians, offers to the notice of the Profession one of the parts in solution, under the name of Liquor Opii Sedativus, as a sedative medicine of the greatest importance.

A most interesting trial for child murder came on at the Warwick Assizes, on Friday morning, the 16th of August last. It terminated in the acquittal of the two young women accused. Dr. Palmer, of Tamworth, who undertook to appear in behalf of the prisoners, will forthwith publish a full account of the trial with elucidatory notes, and general observations on the crime of infanticide, and on the conduct to be pursued by the medical practitioner in solving the important and difficult problem of respiration after birth.

---

Dr. Adams has nearly completed his "Account of Mr. Hunter's Life, Doctrines, and Opinions." The work contains a review of the improvements introduced by his Master, with the state of Surgical Pathology at and before his time.

Mr. Accum has in the press, just ready for publication, "A Practical Essay on Chemical Re-agents or Tests, illustrated by a Series of Chemical Experiments." The work will comprehend a summary view of the general nature of chemical tests, the effects which are produced by the action of these bodies, the uses to which they may be applied, and the art of applying them successfully. A chemical chest, containing the re-agents and apparatus necessary for performing the experiments described in the Treatise, will also be delivered, if demanded, as a companion to the work.

In the Press, Oracular Communications, addressed to Students of the Medical Profession, by Æsculapius.

---

MEDICAL SCHOOL, ST. THOMAS'S AND GUY'S HOSPITALS. The Winter Course of Lectures at these adjoining Hospitals will commence, as usual, the first of October, viz. *At St. Thomas's*, Anatomy and the Operations of Surgery, by Mr. ASTLEY COOPER and Mr. HENRY CLINE; Principles and Practice of Surgery, by Mr. ASTLEY COOPER.—*At Guy's*, Practice of Medicine, by Dr. BABINGTON and Dr. CURRY; Chemistry, by Dr. BABINGTON, Dr. MARCET, and Mr. ALLEN; Experimental Philosophy, by Mr. ALLEN; Theory of Medicine and Materia Medica, by Dr. CURRY and Dr. CHOLMELEY; Midwifery and Diseases of Women and Children, by Dr. HAIGHTON; Physiology, or Laws of the Animal Œconomy, by Dr. HAIGHTON. —These several Lectures are so arranged, that no two of them interfere in the hours of attendance; and the whole is calculated to form a Complete Course of Medical and Chirurgical Instruction. Terms and other Particulars may be learnt on applying to Mr. STOCKER, Apothecary to Guy's Hospital.

MEDICAL THEATRE, ST. BARTHOLOMEW'S HOSPITAL. The following Courses of Lectures will be delivered at this Theatre, during the ensuing Winter. On the Theory and Practice of Medicine by Dr. HUE; on Anatomy and Physiology by Mr. ABERNETHY; on the Theory and Practice of Surgery by Mr. ABERNETHY; on Chemistry and Materia Medica by Dr. HUE; and on Midwifery by Dr. GOOCH. The demonstrations of Anatomy by Mr. STANLEY. To commence on Tuesday, October 1, at two o'clock. Further particulars may be obtained by application to Mr. WHEELER, Apothecary, at the Hospital, or of Mr. Anderson, Me ical Bookseller, West Smithfield.

*St. George's Medical, Chemical, and Chirurgical Schools.*—The Courses will commence the first week in October; 1st. On the Laws of the Animal Œconomy, and the Practice of Physic, by GEORGE PEARSON, M.D. F.R.S. Senior Physician to St. George's Hospital, &c.—2d. On Therapeutics, with Materia Medica and Medical Jurisprudence, by GEORGE PEARSON, M.D. and W. T. BRANDE, F.R.S. Professor, Royal Institution. —3d. On Chemistry, by W. T. BRANDE, F.R.S. Professor of Chemistry, Royal Institution.—4th. On the Theory and Practice of Surgery, by B. C. BRODIE, Assistant Surgeon to St. George's Hospital, &c.—*Note*, Sir E. HOME will continue to give Lectures on Surgery gratuitously to the Pupils of St. George's Hospital.—

A Prospectus may be had at Highley and Son's, Medical Booksellers, 174, Fleet Street.

Dr. ADAMS will commence his Course of Lectures on the Institutes and Practice of Medicine in the beginning of October next.

Mr. CARPUE will commence his Anatomical Lectures, at his Theatre, No. 50, Dean Street, Soho, on Tuesday the 2d of October, 1816.—Further particulars may be known by applying to Mr. Carpue.

Dr. CLUTTERBUCK will begin his Autumnal Course of Lectures on the Theory and Practice of Medicine, Materia Medica, and Chemistry, on Wednesday, October the 2d, at ten o'clock in the morning, at his house, No. 1, in the Crescent, New Bridge Street, Blackfriars; where further particulars may be had.

Mr. WILSON and Mr. BELL will commence their Lectures on Anatomy and Surgery, in Great Windmill Street, on the 1st of October, at two o'clock. Mr. Bell will give a separate Course of Lectures on Surgery in the evening. The anatomical demonstrations will be given by Mr. Shaw, who will direct the Students in their dissections.

Mr. JOHN TAUNTON's Autumnal Course of Lectures on Anatomy, Physiology, Pathology, and Surgery, will commence on Saturday, October 5th, 1816, at eight o'clock in the evening precisely, and be continued every Tuesday, Thursday, and Saturday, at the same hour.—Particulars may be had on applying to Mr. Taunton, 87, Hatton Garden.

DEATHS.— At Southsea, Mr. C. Bingham Hill, Surgeon to the 2nd or Queen's Regiment of Infantry.—Walter Ross Monro, M.D. late Senior Member of the Medical Board at Calcutta.— Charles Taylor, M.D. Secretary to the Society of Arts.— At the house of Mr. Burton, Cross Street, West Smithfield, Dr. Squires, of Ely Place, Holborn; he had been sent for to attend Mrs. Burton, who was in very dangerous labour, and had seated himself on the side of the bed, while Mr. Barnet, Surgeon, was performing the operation; when he suddenly fell upon his face in a fit. Mr. Barnet immediately placed him in a chair and bled him, but he expired without uttering a word.— After a lingering illness, in the forty-third year of his age, Mr. James, Surgeon, Gerard Street, Soho.

